# Editorial: Changing perspectives in speech and language neuropsychology, 1863-2023

**DOI:** 10.3389/fpsyg.2025.1592313

**Published:** 2025-04-11

**Authors:** Paul Eling, Stewart Longman, Frank W. Stahnisch, Pascale Tremblay

**Affiliations:** ^1^Department of Psychology, Radboud University, Nijmegen, Gelderland, Netherlands; ^2^Department of Psychology, University of Calgary, Calgary, AB, Canada; ^3^Departments of Community Health Sciences and History, University of Calgary, Calgary, AB, Canada; ^4^École des sciences de la réadaptation, Université de Laval, Québec, QC, Canada

**Keywords:** aphasia, cerebral localization, founders of neuropsychology, history of neuroscience, holistic perspectives, psycholinguistics, scientific paradigms, speech and language disorders

## Introduction

**Figure 1:**
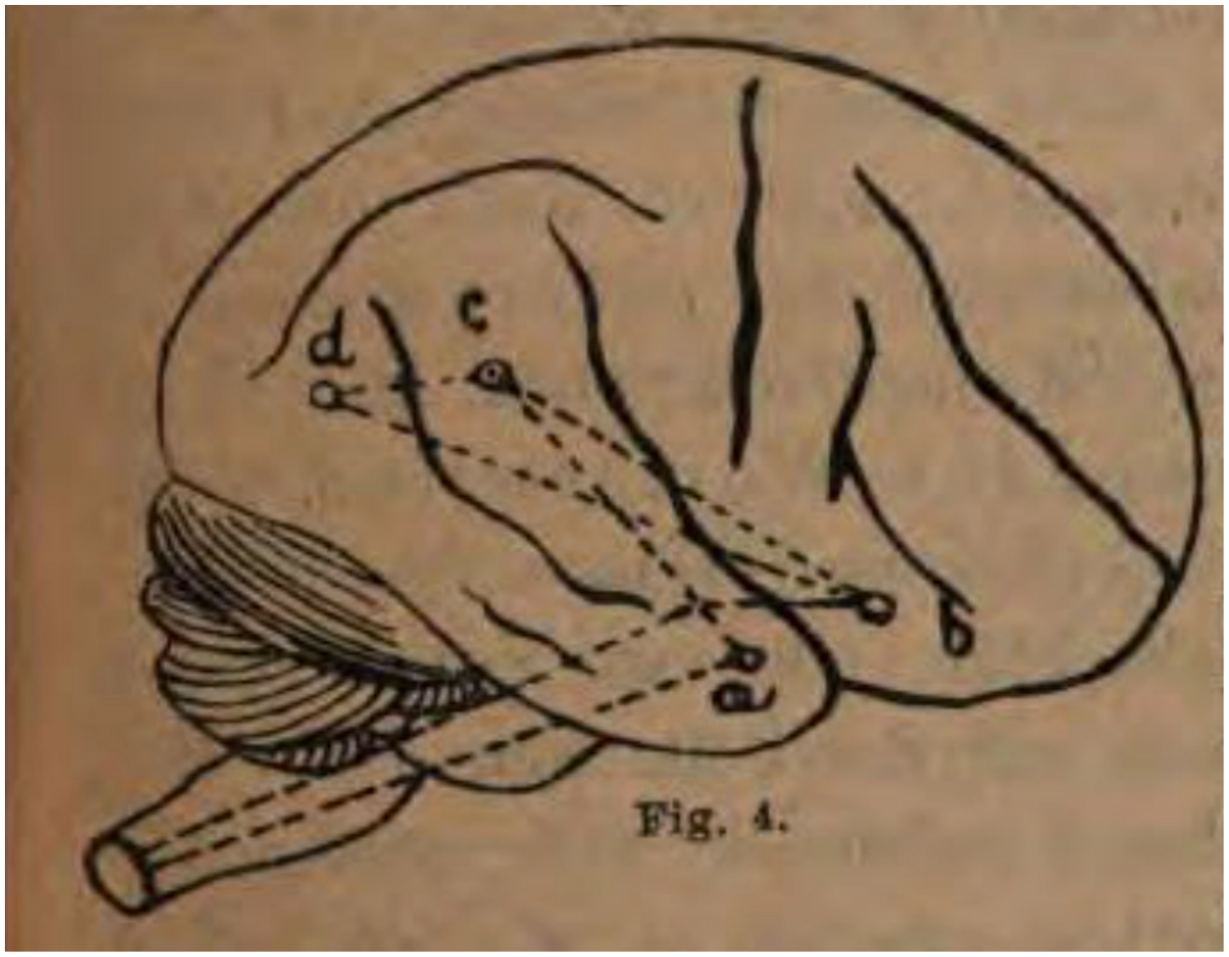
Wernicke (1874), *Der Aphasische Symptomenkomplex. Eine psychologische Studie auf anatomischer Basis*, 23. Sketch © Public Domain.

There have been wide and fundamental changes in the field of speech and language neuropsychology since the publication of Paul Broca's (1824–1880) epoch-making work on “*aphasie*” (*siège du langage articulé*) in 1863 (Broca, 1863). This Research Topic surveys the efforts to understand the relationship between human behavior and brain function with respect to language, cognition, and memory with a focus on activities from the 1860s to 1960s in Europe and North America. The reviewed period begins with the groundbreaking work of Broca in France, John Hughlings Jackson (1835–1911) in Great Britain, and Carl Wernicke (1848–1905) in Germany to identify the neuropathological sources of selective impairments in language (Figure 1) (Levelt, 2013).

Efforts continued throughout the second half of the 19th century, leading to increased activity in the wake of the two World Wars. One hundred years later, interest resurged in the earlier ideas when new approaches were initiated by individuals such as Wilder Penfield (1891–1976) in Canada (Penfield, 1949) and Norman Geschwind (1926–1984) in the United States (Geschwind, 1970). Historiographical approaches to the understanding of these efforts have considered the applied models and metaphors of speech and language neuropsychology, methodological approaches in the clinic and laboratory, as well as on the status of evidence, the flow of ideas and people, along with interdisciplinary exchanges with anthropology, education, linguistics, medicine, and sociocultural contexts (Eling, 1994).

The scholarly collaboration showcased in this Research Topic has generated novel insights and stimulating perspectives on the emergence of speech and language neuropsychology. For instance, the twenty contributing authors have investigated phases and events of the long-standing debate between localizationists and holists in the field of neuropsychology. They have analyzed how and why new concepts and theories have emerged, including for clinical and rehabilitation purposes (Stahnisch and Hoffmann, 2010). Furthermore, the limits imposed by certain models on basic and clinical research since Broca's and Wernicke's times were investigated (Tremblay and Dick, 2016). By bringing scientific authors and humanistic researchers into interaction, this Research Topic has offered unique perspectives and historical case studies that can advance our understanding of neuropsychology, aphasiology, and behavioral neuroscience, while rising above the boundaries between neurological diagnostics, behavioral assessments, clinical applications, and entrenched ways of knowing. This domain of academic research continues to prosper, especially since the increasing uses of modern neuroimaging techniques. The advancement of neuropsychological and cognitive neuroscience research are gaining increasing recognition in the wider medical and rehabilitation community, while actively changing the disciplinary boundaries existing in neurology, psychiatry, psycholinguistics, and clinical psychology (Finkbeiner et al., 2016).

## The individual contributions to this Research Topic

The eight articles included in this special issue cover the development of the field of speech and language neuropsychology over a period of 160 years, such as in the contribution by Tremblay and Brambati (Université Laval, Québec City) and Brambati (Centre de Recherche de l'Institut Universitaire de Gériatrie de Montréal), who examine how 19th-century conceptions and analyses, based in the study of neurological disorders, were transformed through recent studies in the neurobiology of speech and language in relation to physiological insights into neural architecture. The study by Longman and Schwartz (University of Calgary and Dalhousie University, Halifax) investigates the historical conceptualization of “foreign accent syndrome” following brain trauma or due to psychiatric illnesses since the end of the 19th century.

Several of the articles also examine the beginning of the 20th century, which was a time of the interdisciplinary formation of the neurosciences, while the casualties of World War I gave rise to many new insights into the development of speech and language disorders due to the isolated brain lesions in war veterans due to gunshot, shrapnel, and bayonet wounds. Stahnisch's (University of Calgary) contribution delves into Kurt Goldstein's (1878–1965) and Adhémar Gelb's (1887–1936) clinical and psychological works based on war veterans and contrasts these with Norman Geschwind's and his American pupils' positioning toward holistic and localizational perspectives, as they formed the modern-day problem basis in the neurology of aphasia and speech neuropsychology throughout World War II and into the postwar period (Finger, 1994).

Most of the contributions implement localized examples and theoretically focused analyses, placing them in general thematic contexts. The article by Leblanc (McGill University), for example, offers a case study comparing the work of the Russian psychologist and anthropologist Alexander Luria (1902–1977) from the 1930s−1950s, on the acquisition, expression, and loss of articulated and written speech, with the elucidation of the structure-function relationships of the brain by the doyen of Canadian neurological surgery, Wilder Penfield, as he applied electrocortical stimulation techniques in the operation theaters of the Montreal Neurological Institute. Benso et al. (Universities of Trento, Genoa, Geneva, the University of Applied Sciences and Arts of Southern Switzerland in Manno, and the Associazione Neuroscienze Cognitive Clinica Ricerca Intervento at Genova) critically examine the evolution of cognitive modularity and address the challenges of combining foundational theories with empirical findings and theoretical advances. The research by Persichetti et al. (National Institutes of Health and University of California at Los Angeles) shows that categories of abstract concepts (such as emotions, social roles, and mental states) can be formed spontaneously by participants, without requiring explicit instructions or previous judgments about these categories. Alexander et al. (Indiana University Bloomington) analyze the phenomenon of inner speech in the daily lives of people with aphasia, emphasizing the age-dependence of the phenomenon over frequent reports among aphasic test persons and in the existing literature. Finally, Phillips (National Institute of Advanced Industrial Science and Technology, Tsukuba, Japan) focuses on the universality of the Language of Thought hypothesis, emphasizing that psychological category development ensues from a universal mapping principle which connects symbolic and non-symbolic representational formats of cognition and visual perception. Overall, this *Frontiers in Psychology* Research Topic highlights exciting new perspectives and enriches our understanding of the relationship between brain and language. By bridging theoretical insights and empirical findings, providing fresh perspectives on methodological approaches, as well as historical depth, the contributions presented here significantly advance our knowledge and will inform current debates as well as guide future research in the broad field of behavioral and cognitive neuroscience.
